# The Influence of Socioeconomic Status on the Prognosis and Profile of Patients Admitted for Acute Heart Failure during COVID-19 Pandemic: Overestimated Aspects or a Multifaceted Hydra of Cardiovascular Risk Factors?

**DOI:** 10.3390/healthcare9121700

**Published:** 2021-12-08

**Authors:** Radu-Stefan Miftode, Irina-Iuliana Costache, Petru Cianga, Antoniu Octavian Petris, Corina-Maria Cianga, Minela-Aida Maranduca, Ionela-Larisa Miftode, Daniela Constantinescu, Amalia-Stefana Timpau, Adrian Crisan, Ovidiu Mitu, Mihai Stefan Cristian Haba, Celina-Silvia Stafie, Ionela-Lacramioara Șerban

**Affiliations:** 1 Department of Internal Medicine I (Cardiology), Faculty of Medicine, University of Medicine and Pharmacy “Gr. T. Popa”, 700115 Iasi, Romania or radu-stefan.miftode@umfiasi.ro (R.-S.M.); amalia-stefana-v-darie@d.umfiasi.ro (A.-S.T.); crisanadrian93@yahoo.com (A.C.); ovidiu.mitu@umfiasi.ro (O.M.); mihai.haba@umfiasi.ro (M.S.C.H.); 2 Department of Immunology, Faculty of Medicine, University of Medicine and Pharmacy “Gr. T. Popa”, 700115 Iasi, Romania; petru.cianga@umfiasi.ro (P.C.); ccianga@hotmail.com (C.-M.C.); dconstantinescu_ro@yahoo.com (D.C.); 3 Department of Morpho-Functional Sciences (II), Faculty of Medicine, University of Medicine and Pharmacy “Gr. T. Popa”, 700115 Iasi, Romania; minela.maranduca@umfiasi.ro (M.-A.M.); ionela-lacramioara.serban@umfiasi.ro (I.-L.Ș.); 4 Department of Infectious Diseases, Faculty of Medicine, University of Medicine and Pharmacy “Gr. T. Popa”, 700115 Iasi, Romania; larisa.miftode@yahoo.com; 5 Department of Preventive Medicine and Interdisciplinarity, Faculty of Medicine, University of Medicine and Pharmacy “Gr. T. Popa”, 700115 Iasi, Romania; celina.stafie@umfiasi.ro

**Keywords:** socioeconomic status, heart failure, healthcare, cardiovascular risk factors, rural area, low income

## Abstract

*Background*: Heart failure (HF) is a complex clinical syndrome that represents a great burden on public health systems due to its increased prevalence, disability and mortality rates. There are multiple triggers that can induce or aggravate a preexisting HF, socioeconomic status (SES) emerging as one of the most common modifiable risk factors. Our study aimed to analyze the influence of certain SES indicators on the outcome, clinical aspects and laboratory parameters of patients with HF in North-Eastern Romania, as well as their relationship with other traditional cardiovascular risk factors. *Methods*: We conducted a prospective, single-center study comprising 120 consecutively enrolled patients admitted for acute HF. The evaluation of individual SES was based upon a standard questionnaire and evidence from official documents. *Results*: the patients’ age ranged between 18 and 94 years; Out of 120 patients, 49 (40.8%) were women and 71 (59.2%) were men, residing in rural 59 (49.2%) or urban 61 (50.8%) areas. 14.2% were university graduates, while 15.8% had only attended primary school. The majority of the patients are or were employed in the service sector (54.5%), followed by industry (29.2%) and agriculture (20%). The mean monthly income was 306.1 ± 177.4 euro, while the mean hospitalization cost was 2471.8 ± 2073.8 euro per patient. The individual income level was positively correlated with urban area of residence, adequate household sanitation facilities and healthcare access, and negatively associated with advanced age and previous hospitalizations due to HF. However, the individual financial situation was also positively correlated with the increased prevalence of certain cardiovascular risk factors, such as arterial hypertension, anemia or obesity, but not with total cholesterol or male gender. Concerning the direct impact of a poor economic status upon prognosis in the setting of acute HF, our results showed no statistically significant differences concerning the in-hospital or at 1-month follow-up mortality rates. Rather than inducing a direct impact on the short-term outcome, these findings concerning SES indicators are meant to enhance the implementation of policies aimed to provide adequate healthcare for people from all social layers, with a primary focus on modifiable cardiovascular risk factors.

## 1. Introduction

The prevalence of heart failure (HF) shows a rising trend globally, as is the number of people presenting with various risk factors that can trigger an acute HF or decompensate a preexisting one. There is strong evidence to support the association between high mortality rates and cardiovascular diseases (CVD) with more than 10 million deaths worldwide [1], 80% occurring in less-developed countries. The key point of these data is the permanently growing incidence of CVD (and particularly HF) in low or middle-income countries, while in the developed economies the trend is slightly downwards [2].

These figures can be directly related to the global population growth or to local particular epidemiological features, but also to the emergence of new diagnostic techniques and improved screening programs able to detect the so-called subclinical, silent HF. Although, from a medical perspective, the financial cost should be a secondary aspect, we still have to consider the economic impact of HF, which is estimated in various recent studies at tens of billions of dollars, with doubled HF-associated costs projected for 2030 [3]. Paradoxically, although it is a source of significant mortality, the incidence and prevalence of HF are directly correlated with the increase in life expectancy among the general population, even more as HF represents the endpoint syndrome of multiple cardiovascular pathologies. The majority of the pathophysiological processes involved in the development of HF are modifiable throughout the disease course, and every external influence (concerning either lifestyle or medical therapy) can disrupt the chain of events, thus directly affecting both the initial outcome and the long-term prognosis of HF.

In this context, the impact of socioeconomic inequalities upon the prevention, diagnosis and therapeutic approach of HF represents a major and persistent issue for many public health systems. It is well established that socioeconomic status (SES) is a strong predictor of coronary heart disease, which itself represents a leading risk factor for the development of HF [4]. Moreover, SES differences also influence other traditional cardiovascular risk factors that may trigger or aggravate HF, such as hypertension, dyslipidemia or diabetes mellitus [5]. A low level of education is commonly associated with social deprivation, with direct consequences upon behavioral aspects that can influence the course and outcome of CVD and, particularly, HF. These behavioral aspects, such as physical inactivity, smoking, excessive alcohol consumption, low-quality diet and unhealthy eating habits follow a gradient across the socioeconomic spectrum, with the most privileged social groups benefiting from a positive effect in terms of CVD risk [6]. As a result, the increased burden of HF in people with poor SES may be attributed to a plethora of medical, behavioral, and psychosocial risk factors that are more prevalent in vulnerable social groups.

SES comprise several indicators, either specific, individual-related parameters or regionally-based indicators, currently without a consensus on a single or composite best index. SES includes individual level of education, monthly income and professional status, which have demonstrated reliable correlations with prognosis in patients with HF [7]. Locoregional SES indicators are based on the degree of healthcare or educational access, urban versus rural residential environment or even the in-dwelling availability of basic sanitation facilities (e.g., access to tap water, flushing toilets, adequate sewerage). In the same category we need to mention climate, air quality, level of pollution or other environmental issues, which are highly variable and less consistently related with the prognosis of HF.

Concerning large-scale, national indicators, one of the most important parameters of SES is represented by gross domestic product (GDP), or its more precise equivalent, GDP based on purchasing power parity (PPP). The GDP-PPP is widely used in different economic statistics to define poverty thresholds and the actual welfare of a region, and are also an essential component of the calculation of the human development index by the United Nations [8]. The echoes of these national indexes at individual level are represented by minimum or mean monthly wage or pension, overall household income, the percentage of revenues spent on basic necessities, the access to adequate sanitary infrastructure (either public or for individual dwellings), educational and cultural facilities and labor market structure.

The interaction between SES and HF is complex and difficult to assess. Various studies report a 30–50% increase in HF risk due to a poor SES [9,10]. However, an adequate adjustment for other common cardiovascular risk factors (e.g., hypertension, diabetes mellitus, dyslipidemia) has not been comprehensively performed and, thus, the exact risk associated with a deficient SES and its individual contribution in addition to the established risk factors is still unclear. However, healthcare access and medical service providers, and type, doses and adherence to therapies for hypertension, diabetes or ischemic heart disease are usually significantly lower in individuals with a poor SES.

Impaired SES is also associated with a negative prognosis and increased mortality in the presence of a myocardial infarction, either due to delayed presentation, lack of catheterization facilities or due to reduced levels of secondary prevention and coronary revascularization in these patients [11,12]. For example, patients from low-income areas are less likely to undergo a percutaneous coronary intervention within the therapeutic window of an acute myocardial infarction [13] or less likely to benefit from state-of-the-art procedures (e.g., drug eluting stents) or cardiac rehabilitation, which are both essential steps for preventing progression to HF [14,15]. Low-income patients also display a reduced adherence to certain critical medications (statins, antiplatelet agents, diuretics) or to specific behaviors (e.g., avoiding intense physical effort, complete smoking cessation) [16]. Therefore, the occurrence, extent, and progression of myocardial lesions leading to a subsequent ischemic HF are all increased and inversely correlated with the level of socioeconomic development, not only at individual level, but also at a national scale.

Until last year, Romania was considered a middle-income country. From June 2020, the World Bank classified Romania as a high-income country with a gross national income of 12,733 dollars, which is slightly above the threshold for developed country status, calculated at 12,536 dollars, while the preliminary data for 2021 have temporarily downgraded it again, due to the pandemic-related economic crisis [17]. This borderline macroeconomic situation can highlight different SES aspects from both developing and developed countries, thus reproducing more precisely the real impact of SES on the epidemiological, clinical and paraclinical profile of patients admitted for HF. More importantly, the study was conducted in the North-East region of Romania, which ranks among the bottom ten regions in Europe in terms of GDP-PPP, at only 44% of the European Union average in 2019 [18]. The aim of this study was to investigate the presence, distribution and relationship between certain traditional risk factors, clinical and laboratory findings and outcome for hospitalized patients with HF and their SES.

## 2. Materials and Methods

### 2.1. Study Design and Population Characteristics

We conducted a prospective study that included 120 consecutively-enrolled patients who were admitted for acute or decompensated chronic HF in “St. Spiridon” Emergency Clinical Hospital in Iasi, in the North Eastern region of Romania, between January 2021 and May 2021.

We performed several comparative analyses aiming to outline a local HF epidemiological profile by assessing:

1. The influence of certain socioeconomic indicators on the outcome, clinical aspects, laboratory findings and hospitalization costs in patients with HF.
2. The interdependence between poor SES (translated as low income, poor educational status, specific environmental issues) and the presence of other traditional cardiovascular risk factors (e.g., hypertension, diabetes mellitus, dyslipidemia, tobacco use).
3. The coexistence of several indicators suggestive for a poor SES and how they mutually influence each other and the outcome of HF.
4. The relationship between non-modifiable risk factors (i.e., age, gender) and modifiable SES-related parameters in patients with HF from the North Eastern region of Romania.

We included all hospitalized patients presenting with de novo acute HF or with an episode of acute decompensation of a pre-existing HF with the following additional inclusion criteria:

- suggestive clinical syndrome (dyspnea, peripheral oedemas, pulmonary crackles), according to internationally validated Framingham criteria;
- specific echocardiography findings (dimensions, systolic or diastolic dysfunction);
- high-levels of sensitive cardiac biomarkers, such as amino-terminal pro-brain natriuretic peptide (NT-proBNP), cardiac troponin I (cTnI) or the novel sST2;
- the presence of documented comorbidities that represent a well-known etiology for HF (e.g., prior myocardial infarction, valvular pathology).

The exclusion criteria were refusal to undergo a complete cardiological evaluation, the presence of pathological conditions that prevented a correct clinical or echocardiography examination (e.g., thoracic surgery, extreme obesity, severe osteo-articular chest pain), the presence of neuro-psychiatric pathologies that rendered a correct anamnesis difficult, or disagreement with the standard written consent procedure.

The study design was approved by the local ethics committees of both the Grigore T. Popa University of Medicine and Pharmacy (no.9537/2020) and St. Spiridon Emergency Clinical Hospital (no.41/2020) boards, conducted according to the ethical guidelines of the 1975 Declaration of Helsinki Principles, most recently revised in 2013. All patients personally signed an informed consent form in order to participate in this study.

### 2.2. Study and Laboratory Investigations

We analyzed several parameters recorded in the patients’ hospital files. Demographic characteristics (including age, gender), full medical history, particular behavioral conditions (tobacco use, toxic exposure, alcohol abuse-defined as >2 standard drink for men and >1 for women), clinical features (underlying diseases, specific signs and symptoms) were all thoroughly assessed. Some important comorbidities (e.g., COVID-19, diabetes mellitus, obesity, kidney failure) were either previously recorded or newly diagnosed at admission. In order to avoid certain bias, statistical processing only included anthropometric indices such as weight, height and body mass index, as well as blood pressure or glycemia values measured at admission. For example, obesity was established in all patients presenting at admission as a body mass index (BMI) ≥30 kg/m^2^, while overweight was defined by a BMI of between 25 and 29.9 kg/m^2^. Systolic function impairment was assessed by echocardiography (GE Vivid™ V7 ultrasound device by General Electric, Boston, MA, USA) using a cut-off value of <40% for the left ventricle ejection fraction (EF), thus defining HF with reduced EF. The diastolic dysfunction description was based on the three established echocardiography patterns: impaired relaxation, pseudo-normalization and restrictive pattern. Novel biomarkers used for the assessment of HF, such as ST2, were separately analyzed by enzyme-linked immunosorbent assay (ELISA) based kits (Abcam, Cambridge, UK). At admission, all patients were tested for SARS-CoV-2 infection, using standard real-time polymerase chain reaction tests.

### 2.3. Socio-Economic Variables

SES was evaluated by means of a standard questionnaire. The questions were conceived in an objective manner and addressed the fundamental parameters of SES: economic status (monthly income, occupation, employment sector), educational degree (focused on graduate studies), marital status and living conditions (area of residence, basic housing facilities, access to public health system).

In the descriptive statistics, living alone status comprises divorced, widowed or single. Poor education status refers to patients who only attended primary school, while higher education comprises patients with university degrees. Poor housing comprises dwellings without basic sanitation facilities, such as running water or flushing toilets. Poor healthcare access includes the absence of a general practitioner in the area of residence (either rural or urban), as well as the lack of a pharmacy that can provide the patient with the prescribed medication. Low-income threshold has been set at 1386 RON which represents the level of net minimum monthly wage in Romania, roughly equivalent to 282 euros. To facilitate further comparisons, all other amounts are also expressed in euros (1 euro = 4.92 RON, official exchange rate of 25 August 2021).

Routine investigations as part of the standard admission approach (complete blood count, biochemical profile, inflammatory and cardiac biomarkers) and hospitalization costs per patient were compared to the individual SES status, aiming to find relevant correlations.

### 2.4. Statistical Analysis

We performed descriptive statistics in order to highlight the patients’ baseline demographic, socio-economic, clinical and biological characteristics. Categorical variables are presented as numbers and percentages, with continuous variables presented as means ± standard deviations or medians, using the 95% confidence interval in parameter estimation. Differences between subgroups concerning various SES variables were assessed using parametric (independent sample t-test) or non-parametric (Mann–Whitney U) tests, as appropriate. A two-sided *p*-value of <0.05 was considered statistically significant for all of the analyses. In order to assess the correlation between two variables, we used the correlation coefficients (r) of Pearson and Spearman, with the level of statistical significance set at 5%. Additionally, we used linear regression to evaluate the potential relationship between a certain SES variable and a HF prognosis variable, while for the assessment of the correlation between SES status and survival rates we used Mantel–Cox (log-rank) test. Statistical analysis was performed with IBM SPSS version 23, while for the conceptualization of the database we used Microsoft Excel 2013 software (Microsoft, Redmond, WA, USA).

## 3. Results

### 3.1. Baseline Characteristics

Our study included 71 men (59.2%), compared to only 49 women (40.8%) with acute HF, in approximately equal proportions from both urban (50.8%) and rural (49.2%) areas of the North Eastern region of Romania. Acute HF was either de novo (39 cases) or a decompensation of a previously stable, chronic HF (81 cases). Mean age of the analyzed patients was 66.4 ± 15.3 years, ranging from a minimum age of 18 to a maximum age of 94. In-hospital mortality was 17.5% (21 fatalities), while at the 1-month follow-up we recorded an overall mortality rate of 21.7% (26 fatalities). Ninety-three patients were retired (77.5%), the remaining 37 (22.5%) being in the active labor force, of which 19 (15.8%) are currently employed, and only 8 patients (6.7%) were unemployed at the moment of admission.

Regarding the employment sector, the majority of the included patients are or were active in the service sector (45%), followed by industry (29.2%) and agriculture (19.2%). Basic primary school was the only education for 19 patients (15.8%), while university courses had been attended by 17 patients (14.2%), the others having partial (38.3%) or complete (31.6%) secondary education. Familial status is an important aspect for defining individual SES; ff (45.8%) patients have an official marital status and were living with their spouse, while in two cases we recorded an unofficial cohabitation. The rest of the patients were living alone, being either divorced (7.5%), widowed (34.2%) or without any form of relationship (10.8%).

Cardiovascular comorbidities were highly prevalent in our study group, the most common being arterial hypertension (50%), ischemic heart disease (49.2%), arrhythmias (40.8%) and venous thromboembolism (37.5%). Hypertension was significantly more prevalent among women (33 women versus 27 men, *p* = 0.0015), while for the other above-mentioned comorbidities the gender differences were not notable. Diabetes mellitus is an important risk factor for CVD, being found in 22 patients (18.3%). Both infections and previous hospitalizations for HF represent negative predictors for HF, being found in 47.5% and 46.7% of patients, respectively. As a study conducted during the pandemic, the assessment of COVID-19 status was essential: eight patients were associated with coronavirus infection, either previously diagnosed in the last two months or positively tested during hospitalization. None of them were vaccinated.

The high financial burden caused by HF is clearly expressed by the average hospitalization costs (2471.8 ± 2073.8 euro) of the analyzed patients, which are ~8-fold higher than their average monthly income (306.1 ± 177.4 euro).

Detailed data are presented in Table 1.

### 3.2. Cardiovascular-Related Clinical and Paraclinical Profile by Income and Residential Area

Another important aspect of the study was to highlight the relationship between the area of residence and certain parameters involved in the pathophysiology, evolution and prognosis of HF (Table 2). Noteworthy, we observed that patients from rural areas presented a more severe systolic dysfunction, with a significantly lower EF compared to those from urban areas (32.5% versus 21.7%, *p* = 0.009). This aspect could possibly explain the increased need for positive inotropic agents in patients coming from rural areas (10.8% versus 3.3%, *p* = 0.015). On the other hand, arterial hypertension, which is an important etiology for the development of HF, was more prevalent among urban dwellers (31.7% versus 18.3%, *p* = 0.006). Even if infectious pathology was found in a similar proportion within the two groups (25% versus 22.5%, *p* = 0.472), the inflammatory status (expressed as C-reactive protein) was significantly higher in patients from rural areas (*p* = 0.04).

Evaluating the classic risk factors for CVD and, implicitly, for HF, we found no significant variations depending on residence. Lipid profile parameters presented similar serum levels in the two groups, like classic and modern cardiac biomarkers such as NT-proBNP, high-sensitive troponin I (hs-TnI) and sST2, respectively. In the same manner, obesity and overweight affected indiscriminately both rural and urban residents (34.2% versus 36.7%, *p* = 0.751). Special behaviors, which are well-known CVD risk factors, such as tobacco use or alcohol abuse, were more common among patients from rural areas, but without reaching the statistical significance threshold. Contrary, toxic exposure was far more prevalent among urban dwellers, an aspect possibly related to industrialization (*p* = 0.05).

We further observed a significant and positive correlation between rural area of residence and other important SES indicators, such as poor economic status (*p* = 0.006), lack of access to adequate healthcare services (*p* = 0.0001) or dwellings without basic sanitation conditions (*p* < 0.0001). On the other hand, living in an urban center was significantly associated with a higher number of university graduates (10% versus 4.2%, *p* = 0.05), this aspect being an indicator of r reasonable education access and, thus, a more adequate SES.

Despite specific environment-based differences, the outcome of HF was comparable in the two groups, with a very similar in-hospital mortality rate, as well as hospitalization duration or costs, irrespective of area of residence (Table 2).

We also divided the patients according to their net monthly earnings (Table 3). By using the national minimum monthly wage threshold, we noticed important gender associated differences, with a significantly higher proportion of men having monthly income above this value (41 men versus 16 women, *p* = 0.004). Evaluating the classic risk factors for HF decompensation, we found no association between economic status and an increased prevalence of anemia, infections or alcohol abuse, only smoking being more commonly found in patients with a better financial situation (24.2% versus 15.8%, *p* = 0.019).

Concerning cholesterol levels and the prevalence of obese or overweight patients, the economic situation did not play any major role for the analyzed patients, the differences not being statistically significant. The same pattern applies to the levels of cardiac biomarkers, either classic (NT-proBNP, cardiac troponin) or novel (sST2), with very similar serum levels between the two groups. On the other hand, individual economic status directly influenced multiple other SES indicators. Thus, we noticed a significant association between a precarious economic situation and a poor educational profile (*p* = 0.01), the lack of basic sanitary facilities at home (*p* = 0.0001) and living alone (*p* = 0.0006) or in a rural area (*p* = 0.006), while patients with university studies exhibited a significantly better financial status (*p* = 0.0002).

In the light of these results, the next step was to assess the exact correlation (R) between the whole spectrum of monthly income and other relevant SES, clinical, biological or echocardiographic indicators (Table 4). We observed direct positive and significant correlations between individual income and urban area of residence, adequate household sanitation facilities and healthcare access, thus proving the mutual interdependence between these heterogeneous components that define SES status. However, we also noticed a direct correlation with certain cardiovascular risk factors, such as tobacco use, arterial hypertension, anemia and increased BMI, but not with total cholesterol or male gender, and an inverse correlation with age or previous hospitalization in the last year due to HF. Very importantly, a higher monthly income was significantly associated with an improved myocardial systolic function, expressed as left ventricle EF and negatively correlated with the systolic pulmonary artery pression, as the expression of pulmonary hypertension.

### 3.3. The Relationship between Individual Income, HF Prognosis and Economic Burden

**Figure 1:**
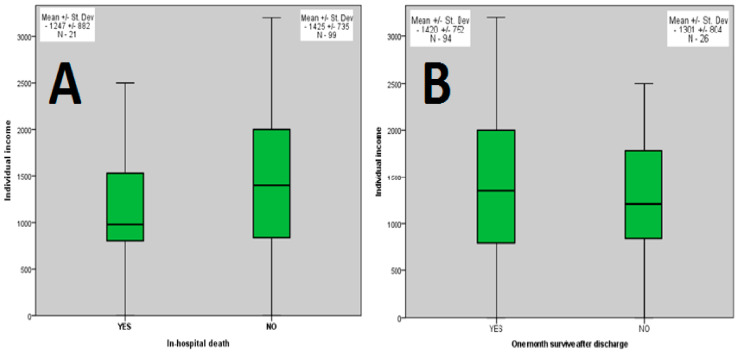
Impact of individual income on in-hospital (**A**) and on 1-month follow-up (**B**) survivability rates in patients with acute HF.

Further, we aimed to assess the potential influence exerted by individual income on the prognosis of acute HF. As depicted in Figure 1, we found no statistically significant income-related differences concerning in-hospital or 1-month follow-up mortality rates.

Moreover, when assessing other relevant SES parameters, we did not notice any important increase in mortality risk that could be related to a specific SES factor (Table 5).

Due to the fact that HF exerts a substantial financial burden on any public health system, it was natural to seek a correlation between the economic status of the patients and their HF-related hospitalization costs. However, we found that the correlation between these variables was weak and not statistically significant r(120) = 0.056, *p* = 0.272 (Table 6). This finding is somehow expected in a country with free and universal healthcare access, where no clear link can be drawn between individual income (and the subsequent contribution to the public health system) and disease-related hospitalization costs, as opposed to countries with a healthcare policy based on private health insurance, where there is an obvious, direct correlation between the type of insurance and the costs of the provided medical services. No significant differences concerning hospitalization costs were observed between patients with acute HF depending on their actual or previous COVID-19 status.

**Figure 2:**
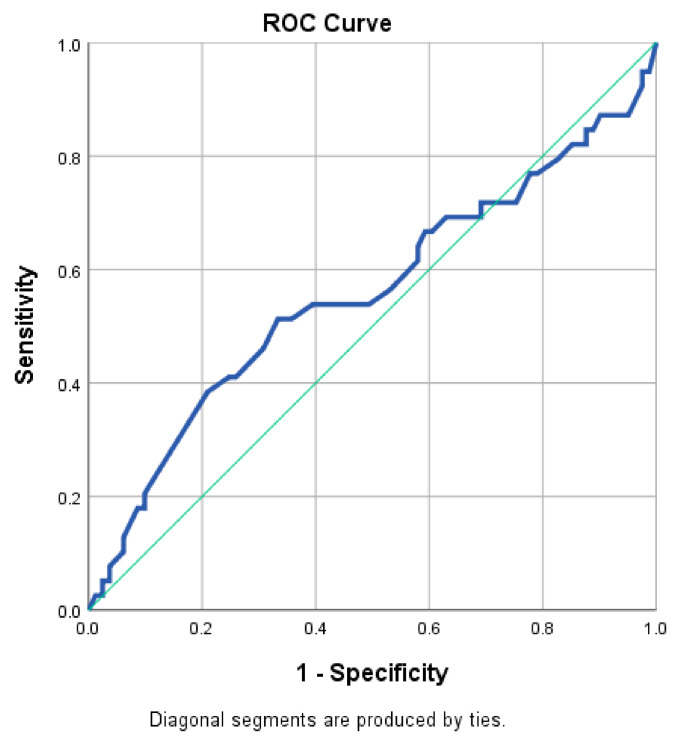
Receiver operating characteristic (ROC) curve for specified income levels.

As mentioned in the initial section, the 120 included patients with acute HF presented symptoms suggestive of either an acute onset de novo HF (31 cases), or an acute decompensation of a previously stable chronic HF (89 cases). We considered it interesting to evaluate the potential influence of poor economic status (expressed as a low monthly income) in the diagnosis of a newly installed acute HF. ROC analysis (Table 7) revealed that the area under the curve (Figure 2) did not exhibit diagnosis performance related to poor financial situation in discriminating between newly diagnosed HF and an exacerbation of a preexisting one (AUC: 0.557). This aspect can be explained by the similar influence exerted by the lack of adequate material means both in the decompensation of a chronic HF (e.g., due to missed doses caused by the inability to buy all prescribed medications, anemia caused by inadequate nutrition, or infections due to unheated, inappropriate housing) and the new onset of acute HF (e.g., due to SES-related risk factors such as certain deleterious dietary habits, inability to perform regular medical check-ups, etc.).

## 4. Discussion

Multiple studies have revealed that socioeconomic deprivation can be an important predictor of HF development and negative outcomes [9,19]. Poor SES can elicit negative effects in HF both directly, via impaired optimal medical access or lack of funds for sustaining a complete standard regimen for HF, and also indirectly, via certain habits and behaviors that can trigger an episode of HF decompensation, such as inadequate diet, high risk of infections or anemia, smoking, excessive alcohol consumption or poor adherence to treatment due to insufficient medical education [20]. In this vast and intricate network of features related to SES, it is difficult to quantify the exact contribution of each negative element, therefore the research spectrum focuses on certain pillars which are easier to systematize: income level, environmental factors and educational attainment, usually with mutual influence on each other. The aim of this study was to find out whether individual SES has any impact on classical cardiovascular risk factors, either demographic, clinical or paraclinical parameters, or even on the outcome of patients diagnosed with acute HF in Romania’s poorest region.

In traditionally high-income countries, individuals with low SES are associated with an increased risk of mortality due to CVD. The controversies concern whether these findings are also applicable to low-income and middle-income countries, which carry about 80% of the total global burden of CVD [20]. Recently promoted to the rank of high-income country, Romania can exhibit characteristics from both categories, also being a highly rural country compared to Western Europe, with a fairly equal distribution between urban and rural population, an aspect which is also mirrored by our study, with 61 patients coming from urban centers and 59 from rural areas.

In our report, the mean age was 66.4 ± 15.3 years (minimum 18- maximum 94), an aspect which is similar to the results from other recent studies that also approached the role of SES in the outcome of CVD [2,21]. Unlike previous studies conducted in the same region which revealed a predominance of women with CVD [22,23], in our study the majority of the patients were male (59.2%). This aspect could be explained by the inclusion of exclusively moderate-severe forms of HF (NYHA III-IV). Even if the total prevalence may be similar between the two genders, severe, late-stage HF is usually more prevalent among men. This difference has been classically explained by the relatively preserved systolic function in women, whose HF etiology is usually arterial hypertension, causing primarily a diastolic dysfunction, whereas ischemia or myocardial infarction represent the most common factors causing HF in men, with a severely impaired EF and a more acute onset and fulminant evolution [24]. Furthermore, SES can directly exert a negative influence on blood pressure, mainly due to dietary habits, especially excessive salt intake, with women of poor SES having significantly greater risk of developing systemic hypertension [6]. This hypothesis was partly confirmed by our findings, the prevalence of hypertension being significantly higher in women and in rural areas, but not among low-income categories.

Traditionally, living in a rural area has been associated with lower life expectancy and impaired access to health care facilities. The Prospective Urban Rural Epidemiology (PURE) trial which included 156,424 persons from 348 urban and 280 rural communities showed that cardiovascular mortality rates as well as total non-fatal cardiovascular events were significantly higher in rural communities (1.71 versus 3.09 events per 1000 person-years, *p* < 0.01 and 4.83 versus 6.25 events per 1000 persons-years, *p* < 0.01, respectively) [25]. Multiple factors may affect this outcome: lack of health care facilities, shortage of practitioners and auxiliary staff, insufficient sanitary education in rural areas, difficult access to urban medical centers due to large distances or transportation costs etc. However, these factors are highly dependent on confounding variables, such as individual economic status, education level and psychosocial aspects [26]. We have also demonstrated this “chain-of-events” paradigm: the patients included in our study who were residing in rural areas presented a lower monthly income, poorer access to healthcare providers and a lower education status. The reciprocal analysis provided similar results: patients with poor economic status presented a low level of education and were generally living alone in rural settlements, thus closing a vicious socio-economic circle. However, when analyzing the data from rural areas, we need to take into account a sensitive topic: decreased healthcare providers’ adherence to the latest guidelines for the treatment of HF. In a Brazilian study [27], only 62% of rural patients with HF received beta-blockers and only 22% of patients with HF in atrial fibrillation received essential oral anticoagulation, similar data as also being found in rural settings from developed countries such as Germany [28] or Australia [29]. For an even worse picture, women from rural areas had higher odds of declaring never having seen a cardiologist (OR 3.88, 95% CI 1.72–8.72) and never having undergone an echocardiogram (OR 2.86, 95% CI 1.42–5.75) when compared with women from urban centers [25]. In our study, we observed that a poor economic status (more prevalent amongst patients from rural areas) was directly correlated with some echocardiographic parameters suggestive for negative prognosis in HF, such as LVEF and sPAP. Despite the fact that echocardiography remains the gold-standard technique for detection of HF in patients complaining of dyspnea, its reduced availability in rural settings, even in developed countries, raises the issue of finding alternative diagnostic tools. Consequently, the use of laboratory kits containing one or multiple cardiac biomarkers can be such an alternative, as they can be used both as surrogate diagnosis criteria, as well as a “red flag” for initiating empirical HF therapy in symptomatic patients. The use of NT-proBNP has been validated by the Screening To Prevent Heart Failure Study (STOP-HF40), the results being readily available and their interpretation easy [30]. In our study which included only patients presenting acute onset moderate-severe HF, we observed marked increases of NT-proBNP levels, without variations concerning area of residence or income level of patients, useful in the diagnosis of HF in rural areas with minimal technical facilities.

A possible alternative to natriuretic peptides could be represented by a novel cardiac biomarker, such as ST2, the latter being also a valuable predictor of mortality and adverse outcomes in patients with HF, as previously revealed in our recent studies [31,32]. In remote locations or in circumstances when echocardiography is unavailable, these new biomarkers can be used either independently or in a multi-marker battery test with NT-proBNP, adding diagnosis and prognosis value when compared to NT-proBNP alone. Moreover, the biomarkers’ value in predicting subclinical HF in patients with preserved LVEF (HFpEF) is also highlighted in a recent review, showing that elevated serum levels of both NT-proBNP and ST2 are suggestive for atrial remodeling, which is often a preliminary morphological step before clinically manifest ventricular dysfunction [33]. Our results show that sST2′s serum levels are very well corelated both with the symptomatology (expressed as dyspnea of NYHA III/IV class), echocardiographic aspects suggestive for HF, and NT-proBNP levels, but independent in relation to classical SES indicators.

Evidence from the literature highlights that individuals with a poor economic situation purchase low quality food and spend their scarce resources on low-cost fast food, saturated fats, and cheap sweets, with only a fraction dedicated to healthy foods, such as vegetables, fresh fruits, fish or lean meat. A low monthly income also leads to the preferential orientation towards canned foods, consisting mainly of low-quality ingredients, high amounts of salt or other preservatives and a high proportion of saturated fats [6]. These habits apply also to drinking pattern: poor SES is associated with high consumption of beer or sweetened beverages, whereas individuals with solid finances usually consume wine (especially red wine), a behavior that, in moderate amounts, is well known for its beneficial effect on CVD health [34]. Despite their proven negative role in HF or other CVD, their impact is difficult to be quantified individually; rather, they represent pieces of a puzzle which make up the entire picture of the unhealthy lifestyle associated with a poor SES and an increased risk for cardiovascular events.

Low-income may represent both a cause and a consequence of a poor education. In our study, patients attending only basic, primary education usually came from rural areas and earned less than their urban, more educated counterparts. Data from South Korean research shows that low education (≤6 years of schooling) was associated not only with increased risk of cardiac events, but also with higher all-cause mortality [35]. An important contributor to a poorly compensated HF is the strong correlation between general education level and health literacy. Patients with poor sanitary education are usually less compliant with their medication and experience worse outcomes. The association between health literacy and prognosis may be partially explained by a poor comprehension of a disease’s mechanisms and severity, by reluctance to change previous habits or even by the incapacity to fully understand the specific recommendations and drug posology from a medical letter [36,37]. Moreover, people with poor SES are very prone to pseudo-science, to “off the record” non-medical advice and to the “fake-news” phenomenon concerning specific therapies, which can further prevent an early presentation to hospital and adequate treatment, thereby possibly inducing HF decompensation. This aspect is particularly evident during the COVID-19 pandemic period, when less developed countries, with a lower human development index or GDP/capita (mutually interchangeable with a low SES), present lower rates of vaccination and decreased adherence to sanitary preventive measures [38], a similar pattern also being observed in the case of reduced adherence to cardiometabolic medication or increased prevalence of various cardiovascular risk factors amongst individuals with modest SES [39,40]. Our results showed that residents of rural areas presented more severe systolic disfunction (*p* = 0.009) and an increased need for positive inotropic support (*p* = 0.0015) compared to urban dwellers; in this situation, delayed medical presentation or low treatment adherence are commonly incriminated risk factors [20].

Kershaw et al. demonstrated that more than half of the total CVD risk in individuals with low SES was attributable to specific behavioral and behavior-induced risk factors, such as smoking (27.3%), obesity (10.2%), physical inactivity (6.3%), and hypertension (5.3%) [41]. However, state policies can modify this pattern through additional taxes and high excise duties on cigarettes and alcohol. Therefore, usually in developed countries, smoking and/or drinking are expensive habits, more commonly found among those with a high economic status. Our study was no exception, as we found a direct correlation between smoking prevalence and higher income in patients with HF (*p* = 0.019).

Last but not least, low-income not only interferes with the above-mentioned modifiable cardiovascular risk factors, but can also influence other SES-related indicators such as distance to healthcare facilities, higher medical costs, insecurity, rural and urban disparities, and gender inequality, that have been reported as barriers to access to an adequate medical approach, aspects that were pointed out in a recent study [42]. Along the same lines, low dietary, behavioral or pharmacological compliance is a well-known causative factor for poor control of HF, with subsequent decompensations and decreased cardiac event-free periods [43,44].

Despite all these observations, in our study we did not notice any significant differences concerning HF outcome or mortality rates directly associated with a specific SES indicator. A possible explanation is related to the existence of a plethora of SES-related risk factors that indiscriminately affect people from all social layers, and to the coexistence in the same individual of discordant socioeconomic parameters (i.e., higher education but low income or vice versa).

A similar paradigm was also valid for hospitalization costs, as we did not observe any direct correlation between these and individual SES. By including patients with acute HF, which is a complex pathology with a costly approach, we expected similar hospitalization costs/patient, irrespective of their prior SES status.

Given that the study was conducted during the COVID-19 pandemic, the costs of hospitalization may have been influenced not only by the infectious disease per se (as only a fraction of the included patients tested positive for the viral infection, presenting just mild forms in our study), but rather by the indirect, fixed costs, regardless of the presence of the infection (i.e., standard protective equipment, costly PCR kits, special healthcare circuits). These expenses are included in the general hospitalization costs and are not influenced by the severity or outcome of the HF. Even if current literature data emphasize the high costs associated with COVID-19 hospitalization, this aspect refers mainly to cases with severe infection, with COVID-19 as the primary pathology leading to emergency admission [45]. Specifically for HF, there are intriguing data: on the one hand, the number of HF-related hospitalizations has decreased by 30–66% during pandemic period, compared to 2017–2019 [46,47,48], on the other hand, the costs per patient have increased due to severe presentations, requiring advanced supportive care and costly equipment and medication [47]. Even in patients with mild forms of the viral infection, there are additional laboratory tests or explorations, not routinely performed in HF, but highly recommended in COVID-19, such as D-dimers or computed tomography [49], which additionally augment the healthcare financial burden.

### Limitations of the Study

The main limitations were the rather small sample group and the unicentric design of the study. Moreover, despite that the vast majority of the included patients were retired, with generally low official monthly incomes, an important proportion were receiving variable remittances (i.e., financial aid sent by relatives working abroad) that rendered challenging an exact assessment of the individual SES. Another limitation for defining a “low” SES refers to people from rural areas, who, despite having low official revenues, may have lower daily-life costs compared to their urban counterparts. This assumption is based on increased food self-provisioning that is commonly found in rural communities and the high degree of mutual aid (even in terms of medication) that characterizes people coming from small villages in the region where the study was conducted. However, these particular aspects erratically influence SES, without the possibility of being objectively quantified.

## 5. Conclusions

In this study, we tried to outline a relevant socioeconomic profile for 120 consecutively enrolled patients, admitted to a university hospital for acute HF. Our results showed positive and significant correlations between income level and other major cardiovascular risk factors such as tobacco use, arterial hypertension, anemia or increased BMI, but not with total cholesterol or male gender. Moreover, we noticed that certain echocardiographic parameters, with a major role in the prognosis of HF, such as LVEF or sPAP, were correlated with individual income. Another important aspect we noticed was the severity of acute heart failure that induced high hospitalization costs in the majority of patients, irrespective of their monthly income, thus drawing attention to the importance of prevention in addressing this pathology, from both medical and socioeconomic perspectives.

Through these elements which are directly related to an increased risk of HF in developed nations, SES as a whole may represent an important predictor of HF, regardless of the individually assessed SES indicators. Even if we did not observe a direct correlation between a specific SES parameter (i.e., monthly income, low education) and the short-term negative outcome in patients with acute HF, it must be taken into account that acute HF is only the final phase of the pathophysiological continuum that leads to cardiac decompensation. In this regard, an extensive assessment of the wide range of factors that outline the general notion of SES is of utmost importance, especially in borderline economic countries (e.g., Romania and other states with similar GDP/PPP), additional research being required in order to implement these multiple parameters into a reliable risk score that could improve HF risk stratification.

The identification of populations with lower SES and the implementation of local and national public health policies aimed to mitigate healthcare inequities and to raise awareness of the importance of CVD may represent a realistic approach for HF prevention. If the HF is already established, regular medical check-ups and guideline-based therapies must be provided to deprived communities in order to control this severe pathology, to maintain a good quality of life and to avoid decompensation episodes. Additionally, an early and comprehensive approach to modifying certain behavioral aspects at the individual and community level may also be considered to ameliorate the quality of life and survival rates in patients with incipient HF.

## Figures and Tables

**Table 1:** Baseline characteristics.

Variable	Value
Age (years, mean ± SD)	66.4 ± 15.3 years (min. 18–max. 94, CI 95%: 2.8)
Gender (%, men: women ratio)	71 (59.2): 49 (40.8)
Area of residence (%, urban: rural ratio)	61 (50.8): 59 (49.2)
Level of occupation (no, %)	Unemployed 8 (6.7) Employed 19 (15.8) Retired 93 (77.5)
Employment sector (no, %)	Agriculture 23 (20) Industry 35 (29.2) Services 54 (45.8)
Level of education (no, %)	Primary school 19 (15.8) Gymnasium 46 (38.3) High school 38 (31.6) University 17 (14.2)
Familial status (no, %)	Married 55 (45.8) Divorced 9 (7.5) Widowed 41 (34.2) Cohabitation 2 (1.7) Single 13 (10.8)
De novo acute HF (no, %)	39 (32.5)
Left ventricle ejection fraction (%, mean ± SD)	33.8 ± 13.9 (min. 10–max. 61, CI 95%: 2.5)
Arterial hypertension (no, %)	60 (50)
Diabetes mellitus (no, %)	22 (18.3)
Ischemic heart disease (no, %)	59 (49.2)
Arrhythmias (no, %)	49 (40.8)
Venous embolism (no, %)	45 (37.5)
Previous hospitalizations for heart failure during the last 365 days (no, %)	55 (46.7)
Infections (no, %) of which: COVID-19 (no, %)	56 (47.5) 8 (6.7%)
Hemoglobin (g/dL, mean ± SD)	13.1 ± 2.1 (min.7.2–max.18.1, CI 95%: 0.36)
C-reactive protein (mg/dL, mean ± SD)	3.5 ± 4.8 (min.0.1–max.32.8, CI 95%: 0.87)
Total cholesterol (mg/dL, mean ± SD)	161.1 ± 51.9 (min.63–max.331, CI 95%: 9.4)
LDL cholesterol (mg/dL, mean ± SD)	106.6 ± 35.6 (min.33–max.255, CI95%: 8.2)
HDL cholesterol (mg/dL, mean ± SD)	39.9 ± 15.9 (min.12–max.111, CI 95%: 2.9)
BMI (kg/m^2^, mean ± SD)	28.5 ± 7.2 (min.7.8–max.77.1, CI 95%: 1.3)
Average monthly income (euro, mean ± SD)	306.1 ± 177.4 (min.40.8–max.816.3, CI 95%: 32.2)
Average hospitalization cost (euro, mean ± SD)	2471.8 ± 2073.8 (min.585.5–max.17436, CI 95%: 374.9)

**Table 2:** Influence of residence on certain clinical and biological parameters in patients with HF.

Parameter	Rural (*n* = 59)	Urban (*n* = 61)	*p*
Age (years, mean ± SD)	64.3 ± 16.2	68.4 ± 14.4	0.127
Gender (men:women ratio)	34 men:25 women	37 men:24 women	0.736
EDeceased (%)	11 (9.2)	10 (8.3)	0.745
Hospitalization (days, mean ± SD)	11 ± 7.3	10.8 ± 5	0.650
Hospitalization costs (euro, mean ± SD)	2495 ± 2399	2402 ± 1459	0.585
Left ventricle ejection fraction <40% (%)	39 (32.5)	26 (21.7)	0.009
Arterial hypertension (%)	22 (18.3)	38 (31.7)	0.006
Hemoglobin (g/dL, mean ± SD)	13.6 ± 3.4	12.7 ± 2	0.114
Infections (%)	30 (25)	27 (22.5)	0.472
C-reactive protein (mg/dL, mean ± SD)	4.25 ± 5.71	2.67 ± 3.55	0.04
Total cholesterol (mg/dL, mean ± SD)	156.1 ± 47.1	166 ± 56.2	0.274
HDL cholesterol (mg/dL, mean ± SD)	38.3 ± 14.8	41.6 ± 16.8	0.250
NT-proBNP (pg/mL, mean ± SD)	8966 ± 7930	8837 ± 9265	0.950
Highly sensitive cardiac troponin I (ng/L, mean ± SD)	1655 ± 7181	1537 ± 6609	0.924
Soluble ST2 (ng/mL)	136.6 ± 116.6	138.9 ± 125.5	0.913
Use of positive inotropic agents (%)	13 (10.8)	4 (3.3)	0.015
Tobacco use (%)	26 (21.7)	22 (18.3)	0.371
Alcohol abuse (%)	40 (33.4)	35 (29.2)	0.240
Exposure to toxic chemicals (%)	16 (13.3)	27 (22.5)	0.05
Obesity and overweight (%)	41 (34.2)	44 (36.7)	0.751
Poor economic status (%)	39 (32.5)	20 (16.7)	0.006
Poor healthcare access (%)	17 (14.2)	2 (1.7)	0.0001
Living in dwellings without basic sanitation facilities (%)	32 (26.7)	6 (5)	<0.0001
Living alone (%)	28 (23.3)	35 (29.2)	0.276
Low education status (%)	12 (10)	7 (5.8)	0.183
University degree (%)	5 (4.2)	12 (10)	0.05

**Table 3:** Influence of poor economic status on certain clinical and biological parameters in patients with HF.

Parameter	Below Minimum Monthly Wage (282 Euro) (*n* = 64)	Above Minimum Monthly Wage (282 Euro) (*n* = 56)	*p*
Age (years, mean ± SD)	66.4 ± 16.1	66.2 ± 14.8	0.951
Gender (men: women ratio)	30 men: 33 women	41 men:16 women	0.004
Deceased (%)	12 (10)	9 (7.5)	0.640
Hospitalization duration (days, mean ± SD)	10.8 ± 6.1	10.8 ± 6.2	0.997
Hospitalization costs (euro, mean ± SD)	2585 ± 2471	2319 ± 1503	0.494
Left ventricle ejection fraction <40%	32 (26.7)	34 (28.3)	0.241
Arterial hypertension (%)	29 (24.2)	35 (29.2)	0.272
Hemoglobin (g/dL, mean ± SD)	13 ± 2.1	13.4 ± 3.5	0.509
Infections (%)	33 (27.5)	25 (20.8)	0.352
C-reactive protein (mg/dL, mean ± SD)	4.1 ± 5.73	2.81 ± 3.52	0.046
Total cholesterol (mg/dL, mean ± SD)	154.3 ± 54.7	167.6 ± 46.8	0.281
HDL cholesterol (mg/dL, mean ± SD)	38.8 ± 14.5	41.8 ± 17.1	0.309
NT-proBNP (pg/mL, mean ± SD)	9431 ± 8466	8136 ± 8798	0.417
High-sensitive cardiac troponin I (ng/L, mean ± SD	1557 ± 6598	1636 ± 7212	0.949
Soluble ST2	152.5	121.1	0.152
Use of positive inotropic agents (%)	10 (8.3)	7 (5.8)	0.625
Tobacco use (%)	19 (15.8)	29 (24.2)	0.019
Alcohol abuse (%)	41 (34.2)	34 (28.3)	0.541
Exposure to toxic chemicals (%)	22 (18.3)	21 (17.5)	0.827
Obesity and overweight (%)	42 (35)	44 (36.7)	0.213
Rural area of residence (%)	39 (32.5)	25 (20.8)	0.006
Poor healthcare access (%)	11 (9.1)	9 (7.5)	0.609
Living in dwellings without basic sanitation facilities (%)	29 (24.2)	8 (6.7)	0.0001
Living alone (%)	44 (36.7)	19 (15.8)	0.00006
Low education status (%)	12 (10)	7 (5.8)	0.01
University education (%)	2 (1.7)	15 (12.5)	0.0002

**Table 4:** Significant correlations between individual income and certain SES, clinical, biological and echocardiographic variables.

Parameters	Individual Monthly Income	
	*p*	R
Urban area of residence	0.01	0.22
Access to running water	0.04	0.18
Living in a house with basic sanitation facilities	<0.01	0.31
Access to a general practitioner	0.03	0.21
Hospitalization in the previous 365 days	0.03	−0.19
Total hospitalization costs	0.72	0.03
Tobacco use	0.04	0.18
Arterial hypertension	0.05	0.13
Anemia (Hemoglobin < 13 g/dL)	0.04	0.18
Total Cholesterol	0.50	0.60
C-reactive protein	<0.01	0.30
Age	0.02	−0.20
Gender (male)	0.06	0.16
BMI	0.04	0.18
LVEF	0.02	0.20
sPAP	0.05	−0.17

BMI—body mass index; LVEF—left ventricle ejection fraction; sPAP—systolic pulmonary artery pressure.

**Table 5:** SES factors influencing mortality risk in acute HF (in-hospital and at 1-month follow-up).

SES Parameter	Deceased (*n* = 26)	Survivors (*n* = 94)	χ^2^	*p*	RR	CI 95%
Rural area of residence	14	45	0.288	0.591	1.20	0.60–2.38
Monthly income less than national minimum wage	16	46	0.257	0.257	1.49	0.70–3.05
Primary education only	5	14	0.285	0.593	1.24	0.56–2.94
Dwelling without basic sanitary facilities	9	29	0.132	0.716	1.15	0.57–2.32
Living alone	13	50	0.08	0.773	0.90	0.45–1.78

χ^2^—chi square test; RR—relative risk; CI 95%—95% confidence interval.

**Table 6:** Correlation between individual income and hospitalization costs.

		Hospitalization Costs	Individual Monthly Income
Pearson Correlation	Hospitalization costs	1.000	0.056
	Individual monthly income	0.056	1.000
Significance (1-tailed)	Hospitalization costs		0.272
	Individual monthly income	0.272	
N	Total = 120 patients	120	120

**Table 7:** AUC detailed analysis: income levels’ capacity in diagnosing newly installed HF.

Test Result Variable(s):				
Area	Std. Error	Asymptotic Sig.	Asymptotic 95% Confidence Interval	
			Lower Bound	Upper Bound
0.557	0.060	0.309	0.439	0.675

## Data Availability

The data presented in this study are available within the article.
